# Infection risk in inflammatory bowel disease patients treated with vedolizumab: a systematic review and meta-analysis

**DOI:** 10.3389/fmed.2026.1806488

**Published:** 2026-06-11

**Authors:** Lidan Zhang, Xiaogang Hao, Yidan Cui, Wenjian Yan, Kun Shang, Xin Zhang

**Affiliations:** 1 College of Acupuncture and Tuina, Changchun University of Chinese Medicine, Changchun, China; 2 College of Pharmacy, Changchun University of Chinese Medicine, Changchun, China

**Keywords:** comparative safety, infection risk, inflammatory bowel disease, meta-analysis, vedolizumab

## Abstract

**Objective:**

To evaluate the infection risk associated with vedolizumab (VDZ) in patients with inflammatory bowel disease and to compare its safety with tumor necrosis factor inhibitors, ustekinumab, and placebo.

**Methods:**

A systematic search of five databases was conducted from inception to December 2025. Infection outcomes were standardized per CTCAE v5.0. Proportion meta-analyses employed random-effects models with Knapp-Hartung adjustment and prediction intervals; comparative safety was assessed by pooling risk ratios. Meta-regression explored sources of heterogeneity.

**Results:**

Sixty-six studies (24,000 patients) were included. The overall pooled infection incidence was 13.08% (95% CI: 9.85–17.18%), with respiratory tract infections being the most common (4.25%). Serious systemic and rare infections were infrequent. No significant difference in infection risk was observed between VDZ and TNF inhibitors (RR = 0.91) or placebo (RR = 1.02). VDZ was associated with a significantly higher risk compared with ustekinumab (RR = 1.63). Meta-regression identified study design and geographic region as significant moderators.

**Conclusion:**

VDZ is associated with a moderate infection risk, primarily consisting of mild respiratory events, and a safety profile broadly comparable to TNF inhibitors and placebo but inferior to ustekinumab. Extreme heterogeneity limits the clinical utility of pooled incidence estimates; comparative risk assessment is essential for therapeutic decision-making.

**Systematic review registration:**

https://inplasy.com/inplasy-2026-6-0022/, identifier INPLASY202660022.

## 1 Introduction

Inflammatory bowel disease (IBD) refers to immune-mediated chronic inflammation of the gastrointestinal tract, consisting mainly of CD and UC. Its pathogenesis is complex, involving intestinal mucosal immune dysfunction, abnormal activation of innate and adaptive immunity, intestinal flora imbalance, genetic predisposition, and environmental factors. The primary therapeutic goals for IBD management are to induce and maintain clinical remission, prevent complications, and improve patient quality of life. With advances in understanding IBD immune pathogenesis, biological agents targeting key immune molecules have gradually replaced conventional non-specific immunosuppressants as the first-line treatment for moderate-to-severe IBD, significantly improving treatment efficacy and patient prognosis.

Vedolizumab (VDZ) is a humanized IgG1 monoclonal antibody targeting α4β7 integrin. It exerts its therapeutic effect by binding to α4β7 integrin on the membrane surface of memory T and B lymphocytes, thereby inhibiting the interaction between α4β7 integrin and mucosal addressin cell adhesion molecule-1 (MAdCAM-1) on intestinal endothelial cells. This blockade prevents lymphocyte trafficking from the peripheral circulation into the intestinal mucosa, reducing local intestinal inflammation without impairing systemic immune function, an intestine-specific characteristic that distinguishes VDZ from other biological agents. Preclinical evidence demonstrates that VDZ reduces the infiltration of CD4+ T cells, CD8+ T cells, and pro-inflammatory cytokines (TNF-α, IL-6, IL-17) into the intestinal mucosa while preserving systemic immune function. VDZ was approved by the US Food and Drug Administration (FDA) in December 2014 and the European Medicines Agency (EMA) in July 2015 for the treatment of moderate-to-severe CD and UC, and has since been widely used in clinical practice. Real-world data indicate that VDZ induces clinical remission in 40–60% of IBD patients and histological healing in 30–45% (1). IBD patients who have failed or are intolerant of anti-TNF-α inhibitors show the most favorable clinical responses to VDZ in real-world settings. The growing clinical application of VDZ has raised increasing concerns regarding potential safety risks, particularly infectious complications (2).

Despite its distinct intestinal-targeting advantages, VDZ is still associated with a certain risk of infectious complications. Existing clinical and real-world studies have reported conflicting results on the incidence of infections in VDZ-treated IBD patients. These discrepancies may be attributed to variations in study design, sample size, baseline patient characteristics, definitions and classification of infectious events, diagnostic criteria for infections, and follow-up duration. Additionally, some studies report a higher infection risk in CD patients compared with UC patients (3), while others fail to identify this disparity. Furthermore, the site-specific infection risks and risk of severe infections in VDZ-treated patients remain unclear, hindering the development of personalized clinical management strategies.

Several systematic reviews and meta-analyses have investigated the safety of VDZ in IBD, but these studies suffer from notable limitations, including a small number of included studies, incomplete coverage of infection subtypes, inadequate subgroup analyses, and limited comparative data with newer biologic agents (4, 5). With the continuous publication of new clinical and real-world data, an updated meta-analysis incorporating additional research findings and rigorous comparative analyses is urgently needed to clarify the VDZ-associated infection risk in IBD patients, characterize subgroup-specific infection risks, and provide evidence for rational clinical medication and safety monitoring. We thus conducted a comprehensive systematic review and meta-analysis of relevant clinical studies to evaluate the overall infection risk, subgroup differences, site-specific infection prevalence, and comparative safety of VDZ relative to other common therapies, aiming to provide a scientific basis for the clinical application of VDZ.

## 2 Materials and methods

This systematic review and meta-analysis was conducted in accordance with the Preferred Reporting Items for Systematic Reviews and Meta-Analyses (PRISMA) 2020 guidelines (6).

### 2.1 Inclusion and exclusion criteria

#### 2.1.1 Inclusion criteria

(1) Study population: Patients diagnosed with IBD (CD or UC) based on internationally recognized criteria (7, 8).
(2) Intervention: Patients receiving VDZ monotherapy or VDZ combination therapy (with immunosuppressants including azathioprine, mercaptopurine, or methotrexate), with clearly reported VDZ dosage, administration route, and treatment duration. Studies with comparator groups (TNFis, UST, placebo) were prioritized.
(3) Outcome measures: Studies reporting infectious events, including overall infection (diagnosed based on clinical manifestations, laboratory tests, or imaging examinations), respiratory tract infection, gastrointestinal infection, skin and soft tissue infection, genitourinary infection, systemic invasive infection, and opportunistic infection. All infection outcomes were standardized according to the Common Terminology Criteria for Adverse Events (CTCAE) version 5.0 (9). The classification table of infections included in the study can be found in Supplementary Table 1.
(4) Study design: Randomized controlled trials (RCTs), prospective or retrospective cohort studies, and case-control studies.
(5) Literature characteristics: Full-text articles published in English, with complete data on the number of infection events and total sample size.

#### 2.1.2 Exclusion criteria

(1) Literature type: Reviews, case reports, commentaries, letters to the editor, conference abstracts, and preclinical studies (*in vitro* or animal studies).
(2) Duplicate publications/overlapping populations: Multiple publications from the same research team or with overlapping study populations, with only the most comprehensive and detailed study retained.
(3) Incomplete data: Studies with missing infection-related data (e.g., number of infections, total sample size) that could not be supplemented even after contacting the corresponding authors.
(4) Specific populations: Studies focusing exclusively on pregnant/lactating women, or patients with severe comorbidities, unless subgroup analysis for these populations was pre-planned.

### 2.2 Search strategy

The search was conducted in accordance with the PICOS framework. Search terms included Medical Subject Headings (MeSH) and free-text words: Vedolizumab, Anti-alpha 4 beta 7 integrin antibody, Inflammatory Bowel Disease, Crohn’s Disease, Ulcerative Colitis, Infection, Infectious complication, Adverse event, Safety, etc. These terms were combined using Boolean operators (AND/OR), and the search strategy was adapted to the specific syntax of each database. The complete database-specific search strings are provided in Supplementary Material 2.

The following electronic databases were searched from inception to December 31, 2025: PubMed, Web of Science, Ovid MEDLINE, EMBASE, and the Cochrane Central Register of Controlled Trials (CENTRAL). Additionally, the reference lists of included studies and relevant systematic reviews/meta-analyses were manually screened to identify additional eligible studies, to minimize the risk of missing relevant literature. EndNote X9.1 software was used to manage search results; duplicate records were first removed using the software’s built-in deduplication function, followed by manual verification.

### 2.3 Data extraction and quality assessment

#### 2.3.1 Data extraction

Two independent researchers (Investigator A and Investigator B) extracted data from all included studies using a standardized data extraction form. Inter-reviewer agreement was calculated using Cohen’s kappa coefficient, with disagreements resolved by consensus or adjudication by a third senior investigator. The extracted data included the following items:

(1) Basic study information: First author, publication year, study location, study design, and follow-up duration.
(2) Patient characteristics: Total sample size, number of CD/UC patients, mean age ± standard deviation (SD) or median age with interquartile range (IQR), sex ratio, disease activity, prior treatment (anti-TNF-α inhibitors or other biological agents), and concomitant immunosuppressant use.
(3) Treatment regimen: VDZ dosage, treatment duration, and comparator therapy details (if applicable).
(4) Outcome measures: Number of overall infections, site-specific infection cases, total sample size, study-defined criteria for infection, and number of severe/opportunistic infections.

For studies with incomplete data, the corresponding authors were contacted via email to request Supplementary Information. Studies for which no response was received within two weeks were excluded due to incomplete data.

#### 2.3.2 Quality assessment

The methodological quality of non-randomized studies (cohort and case-control studies) was independently assessed by two researchers using the Newcastle-Ottawa Scale (NOS) (10, 11). The NOS includes three domains: Study Group Selection (4 points), Group Comparability (2 points), and Outcome Assessment (3 points), with a maximum total score of 9 points. Studies with an NOS score ≥ 6 were deemed high quality and included in the final analysis; those with an NOS score < 6 were excluded due to low methodological quality.

The methodological quality of RCTs was assessed using the Cochrane Risk of Bias Tool 2.0 (RoB 2.0) (12), which evaluates five key domains: randomization process, deviations from intended interventions, missing outcome data, measurement of the outcome, and selection of the reported result. Each domain was rated as low risk, some concern, or high risk of bias. Consistency of quality assessment results was verified between the two researchers; disagreements were resolved through discussion with a third independent researcher. Detailed study-level quality assessment results are presented in Supplementary Table 3.

### 2.4 Statistical analysis

Meta-analysis was performed using R software (version 4.2.2) with the meta and metafor packages. For proportion meta-analyses, infection incidence with 95% confidence interval (CI) was used as the effect size indicator. Normality of raw proportions and four common transformations (log, logit, arcsine, Freeman-Tukey double arcsine) was assessed using the Shapiro-Wilk test for each outcome, and the transformation method yielding the highest p-value was selected to optimize normality (13). All proportion meta-analyses employed restricted maximum likelihood (REML) random-effects models with Knapp-Hartung adjustment to account for small sample sizes and extreme heterogeneity (14). Prediction intervals (95% PI) were calculated to quantify the range of true effect sizes expected in future studies (15).

Statistical heterogeneity was assessed using the Cochrane Q test and I^2^ statistic. A *P* < 0.05 in the Q test indicated the presence of significant heterogeneity, and the I^2^ statistic quantified the magnitude of heterogeneity. Subgroup analyses were conducted to explore potential sources of heterogeneity, including publication year, study design, study scale/organization type, predominant patient age, geographic region (Asia, Europe, North America, Global), and follow-up duration. Meta-regression was performed to quantify the contribution of multiple covariates (follow-up duration, region, etc.) to observed heterogeneity.

For comparative safety analyses, risk ratios (RR) with 95% CIs were pooled from head-to-head studies comparing VDZ with TNFis, UST, or placebo. Publication bias was evaluated using funnel plots and Egger’s linear regression test. The trim-and-fill method was applied to correct the pooled effect size and assess the impact of publication bias if significant bias was detected. All statistical tests were two-tailed, with a significance level of α = 0.05 (95% confidence level).

## 3 Results

### 3.1 Basic characteristics and quality evaluation of included studies

**FIGURE 1:**
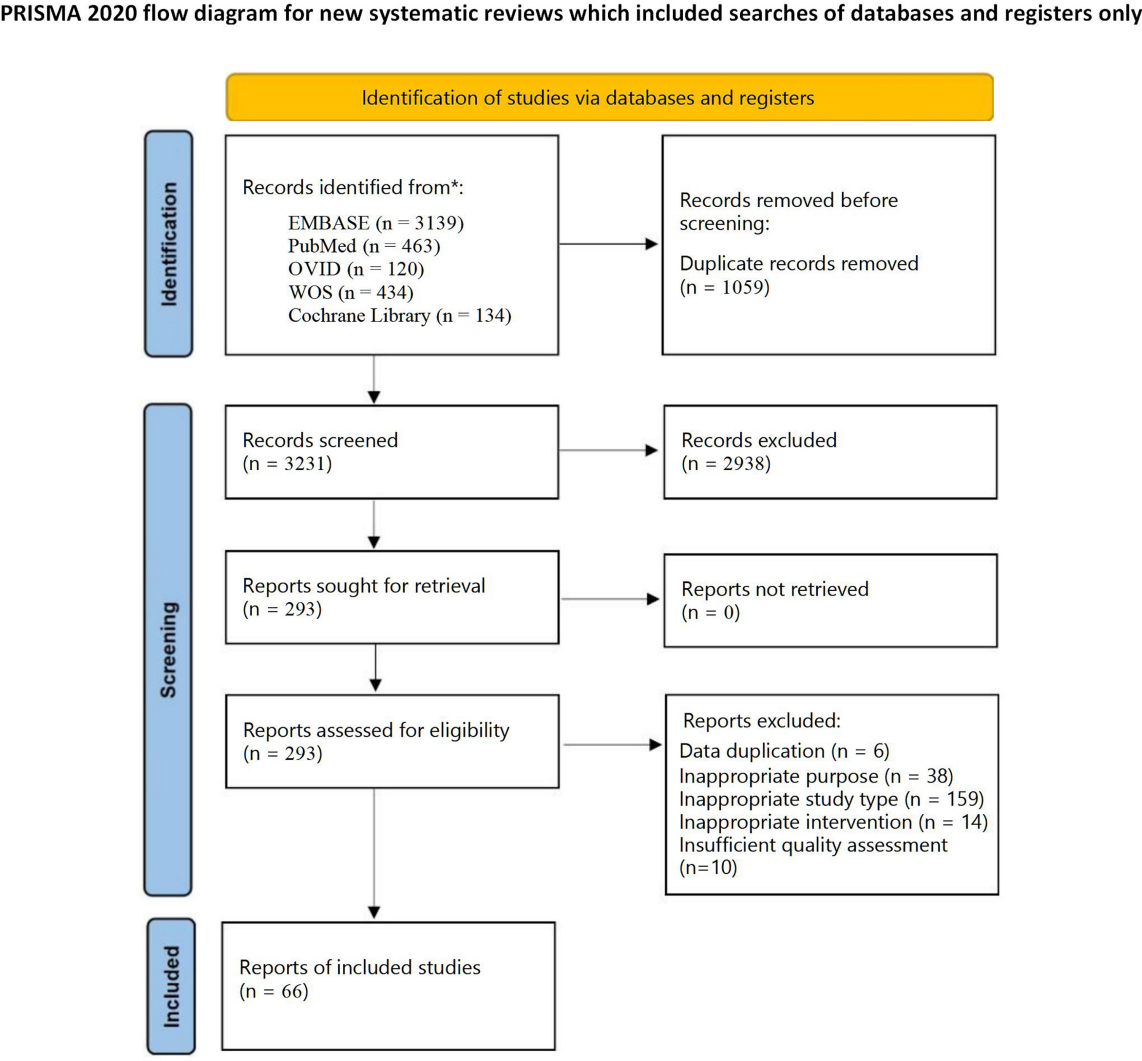
Flow chart of study selection process. From Page et al. (6).

A total of 4,290 records were initially retrieved, and 66 studies were finally included in this meta-analysis after stepwise screening (Figure 1) (16–81). Inter-reviewer agreement, assessed by Cohen’s κ, was 0.91 (95% CI, 0.89–0.93) for title/abstract screening and 0.85 (95% CI, 0.78–0.92) for full-text review. The PRISMA flow diagram details the number of records identified, screened, assessed for eligibility, and included, with specific reasons for exclusion at each stage. A small number of studies were excluded from individual subgroup analyses due to incomplete data for specific stratification factors.

The 66 included studies involved a total of 24,000 IBD patients treated with VDZ, including 12,696 with CD (reported in 54 studies) and 11,304 with UC (reported in 54 studies). Most patients received the standard intravenous VDZ regimen (300 mg at week 0, week 2, week 6, and then every 8 weeks thereafter). 18 studies included a TNFi comparator group, 12 studies included a UST comparator group, and 4 studies included a placebo comparator group.

Quality Assessment Results: All included non-randomized studies (58 cohort and case-control studies) were assessed using the Newcastle-Ottawa Scale (NOS). All studies had an NOS total score ≥ 6, indicating an overall high methodological quality of the included non-randomized studies.

The 8 RCTs were assessed using the Cochrane Risk of Bias Tool 2.0. 4 studies were rated as “low risk” in all domains, yielding an overall risk of bias of “low risk.” The other 4 studies were rated as “some concerns” in at least one domain, leading to an overall risk of bias of “some concerns.” These concerns were predominantly in the domains of “deviations from intended interventions,” “measurement of the outcome,” and “missing outcome data.” No study was rated as “high risk” in any domain.

Detailed baseline characteristics of the studies, patient baseline data, treatment regimens, outcome measures, and quality evaluation results are summarized in Supplementary Table 4.

### 3.2 Overall infection risk in VDZ-treated IBD patients

**FIGURE 2:**
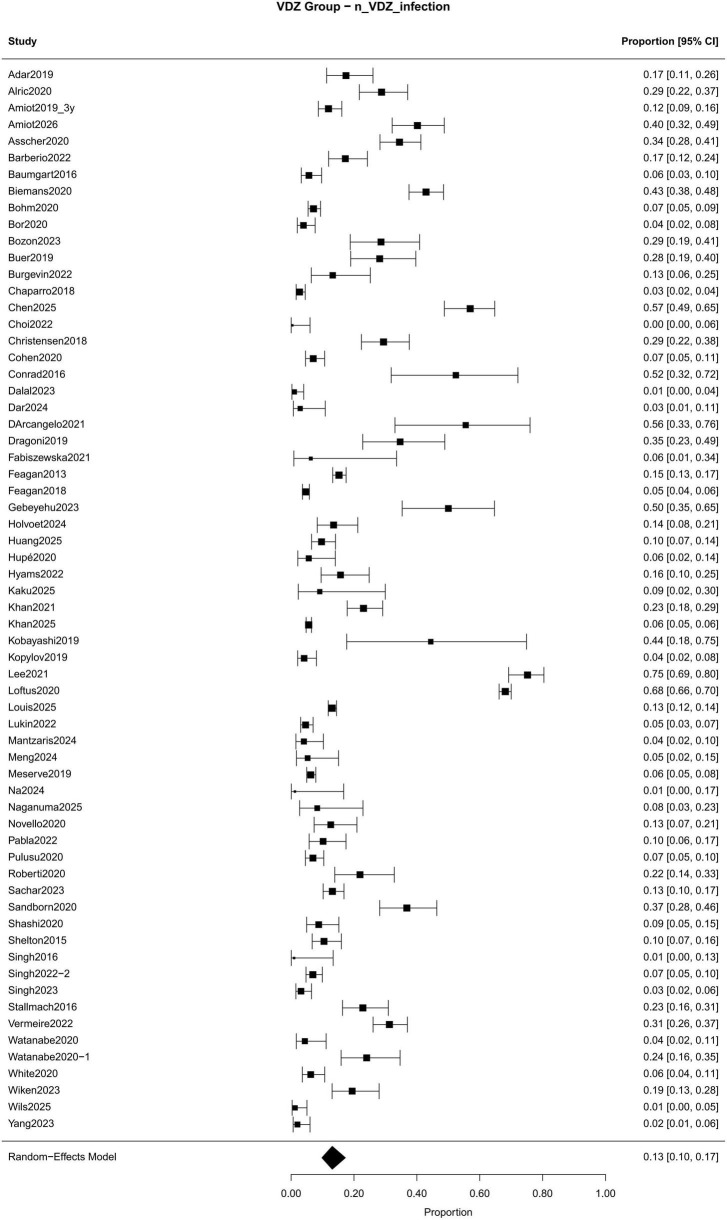
Forest plot of overall infection risk in VDZ-Treated IBD patients.

Meta-analysis of 64 studies involving 24,000 patients revealed an overall infection incidence of 13.08% (95% CI: 9.85–17.18%) in VDZ-treated IBD patients (Figure 2). The Cochrane Q test indicated substantial heterogeneity across studies (*Q* = 4015.22, df = 63, *P* < 0.001, *I*^2^ = 97.85%). The 95% prediction interval was 1.30–63.29%, indicating extreme between-study variability. Sensitivity analysis (sequentially excluding one study at a time) showed the pooled infection incidence ranged from 12.58 to 13.52%, suggesting the results were not driven by a single study.

Publication bias assessment showed an asymmetric funnel plot for overall infection risk (Supplementary Figure 1), and Egger’s test confirmed significant publication bias (*P* = 0.0021). After trim-and-fill adjustment, the pooled infection incidence was 17.08% (95% CI: 12.88–22.29%), which was higher than the unadjusted value, but its 95% CI still overlapped with the original 95% CI, suggesting a limited impact of publication bias on the overall results.

### 3.3 Infection risk in disease-specific subgroups

#### 3.3.1 CD subgroup

**FIGURE 3:**
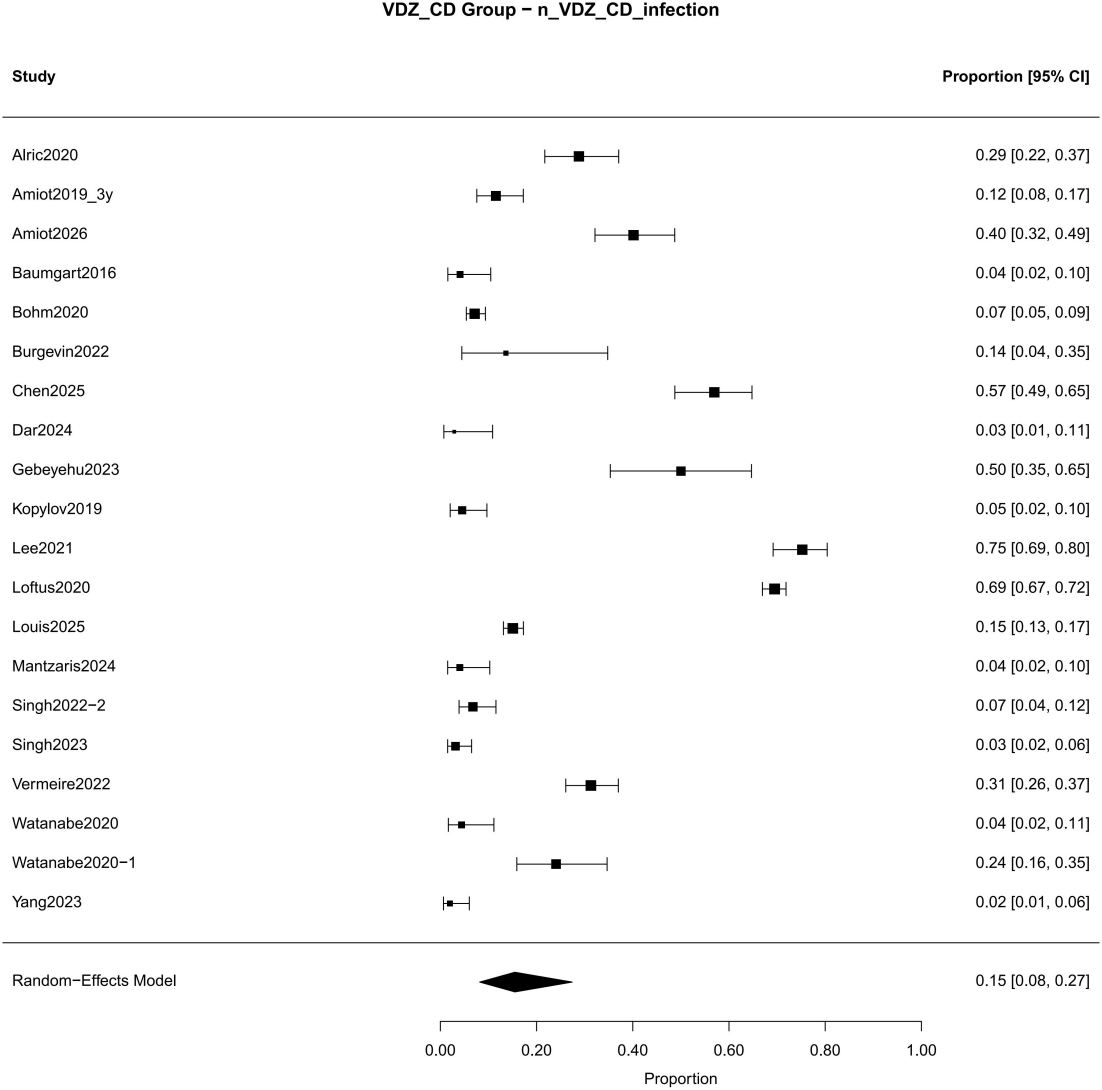
Forest plot of infection risk in CD subgroup.

Twenty studies were included in this subgroup analysis. Meta-analysis showed an overall infection incidence of 15.49% (95% CI: 8.53–26.50%) in VDZ-treated CD patients (Figure 3). The Cochrane Q test indicated extreme heterogeneity across studies (*Q* = 1376.65, df = 19, *P* < 0.001, *I*^2^ = 98.63%). The 95% prediction interval was 0.72–82.20%, indicating extreme between-study variability. Sensitivity analysis showed the pooled infection incidence ranged from 13.67 to 16.97%, suggesting the results were not driven by a single study.

Publication bias assessment showed an asymmetric funnel plot, and Egger’s test confirmed significant publication bias (*P* = 0.0133). After trim-and-fill adjustment, the pooled infection incidence was 16.90% (95% CI: 9.36–28.62%), which was slightly higher than the unadjusted value, but its 95% CI still overlapped with the original 95% CI. Among site-specific infections, respiratory tract infections were the most common (8.61%, 95% CI: 2.92–22.80%; *I*^2^ = 99.23%), followed by digestive system infections (4.48%, 95% CI: 2.77–7.15%; *I*^2^ = 89.11%).

#### 3.3.2 UC subgroup

**FIGURE 4:**
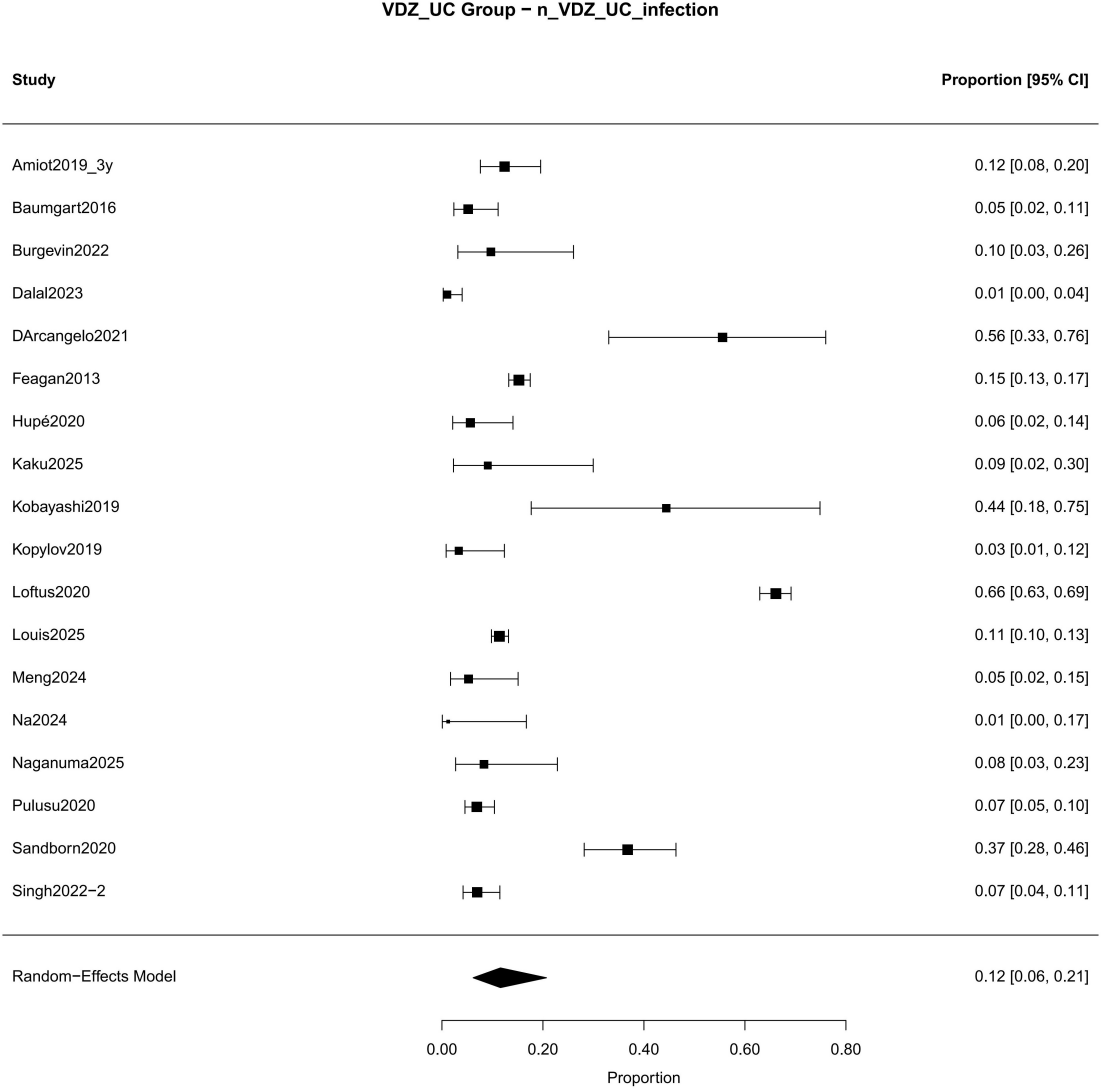
Forest plot of infection risk in UC subgroup.

Eighteen studies were included in this subgroup analysis. Meta-analysis showed an overall infection incidence of 11.59% (95% CI: 6.47–19.91%) in VDZ-treated UC patients (Figure 4). The Cochrane Q test indicated substantial heterogeneity across studies (*Q* = 987.13, df = 17, *P* < 0.001, *I*^2^ = 97.68%). The 95% prediction interval was 0.81–67.70%, indicating extreme between-study variability. Sensitivity analysis showed the pooled infection incidence ranged from 10.03 to 13.14%, suggesting the results were not driven by a single study.

Publication bias assessment showed no significant funnel plot asymmetry, and Egger’s test did not indicate significant publication bias (*P* = 0.1289). After trim-and-fill adjustment, the pooled infection incidence was 14.98% (95% CI: 8.37–25.35%), which was higher than the unadjusted value, but its 95% CI still overlapped with the original 95% CI. Respiratory tract infections were also the most common site-specific infection (13.05%, 95% CI: 5.69–27.20%; *I*^2^ = 98.86%), followed by digestive system infections (2.95%, 95% CI: 1.96–4.43%; *I*^2^ = 55.70%).

### 3.4 Site-specific infection risk

Site-specific infection risks are summarized in Table 1. It is important to note that site-specific estimates are based on different subsets of studies and are not necessarily additive components of the overall infection incidence.

**TABLE 1:** Site-specific infection risks in VDZ-treated IBD patients.

Infection type	Number of studies	Pooled incidence (95% CI)	*I*^2^ (%)	95% prediction interval
Respiratory tract	56	4.25% (2.63% - 6.81%)	98.24	0.15–56.40%
Digestive system	56	2.18% (1.58–2.99%)	85.12	0.34–12.63%
Genitourinary system	56	0.73% (0.48–1.10%)	71.54	0.06–8.40%
Skin and soft tissue	56	1.00% (0.67–1.49%)	76.68	0.09–10.53%
Systemic invasive	57	0.56% (0.40–0.77%)	57.88	0.09–3.32%
Nervous system	56	0.35% (0.25–0.48%)	0.00	0.25–0.49%
Musculoskeletal	56	0.33% (0.23–0.46%)	0.00	0.23–0.46%
Ocular	56	0.34% (0.24–0.47%)	0.00	0.24–0.47%
Pathogen-specific	56	0.80% (0.58–1.11%)	60.74	0.14–4.52%
Other	63	1.17% (0.71–1.91%)	94.63	0.03–31.77%

Respiratory tract infections were the most common site-specific infection, with a pooled incidence of 4.25% (95% CI: 2.63–6.81%) and substantial heterogeneity (*I*^2^ = 98.24%). Digestive system infections (2.18%, 1.58–2.99%) and skin and soft tissue infections (1.00%, 0.67–1.49%) also exhibited relatively higher incidences. Systemic invasive infections were rare, with a pooled incidence of 0.56% (95% CI: 0.40–0.77%). Nervous system, musculoskeletal, and ocular infections had extremely low incidences ( < 0.5%) with no significant heterogeneity (*I*^2^ = 0% for all), indicating consistent results across studies.

### 3.5 Comparative safety analysis

#### 3.5.1 VDZ vs. TNF inhibitors

Seventeen studies were included in this comparison. Meta-analysis showed no statistically significant difference in overall infection risk between VDZ and TNF inhibitors (RR = 0.908, 95% CI: 0.668–1.234, *P* = 0.513). Significant heterogeneity was observed (*Q* = 62.30, df = 16, *P* < 0.001; *I*^2^ = 70.92%, tau^2^ = 0.194) (Table 2 and Supplementary Figure 2).

**TABLE 2:** Characteristics of studies included in comparative safety analyses.

Study ID	n-VDZ	n-VDZ-infection	n-control	n_control_infection	Control-intervention
Feagan et al. (43)	1,096	167	424	63	PBO
Feagan et al. (42)	1,434	66	297	13	PBO
Vermeire et al. (76)	275	86	134	46	PBO
Watanabe et al. (77)	79	19	78	11	PBO
Adar et al. (16)	103	18	131	26	TNF
Barberio et al. (21)	145	25	93	15	TNF
Bohm et al. (24)	659	47	607	47	TNF
Bozon et al. (26)	63	18	113	33	TNF
Dalal et al. (36)	195	2	610	48	TNF
D’Arcangelo et al. (39)	18	10	167	54	TNF
Huang et al. (48)	238	23	186	24	TNF
Hupé et al. (46)	71	4	154	6	TNF
Khan et al. (56)	2,747	153	3,914	109	TNF
Lukin et al. (58)	454	21	268	27	TNF
Mantzaris et al. (59)	99	4	63	7	TNF
Meng et al. (60)	57	3	65	10	TNF
Naganuma et al. (63)	36	3	34	2	TNF
Pabla et al. (65)	108	11	104	16	TNF
Roberti et al. (67)	73	16	550	75	TNF
Singh et al. (73)	377	26	377	24	TNF
Singh et al. (74)	221	7	1,030	53	TNF
Alric et al. (17)	132	38	107	12	UST
Amiot et al. (19)	132	53	107	19	UST
Asscher et al. (20)	203	70	207	49	UST
Burgevin et al. (28)	53	7	31	7	UST
Choi et al. (31)	125	0	113	0	UST
Dar et al. (38)	69	2	97	5	UST
Gebeyehu et al. (44)	42	21	83	26	UST
Holvoet et al. (45)	111	15	60	6	UST
Kaku et al. (49)	22	2	29	1	UST
Na et al. (62)	40	0	11	0	UST
Sachar et al. (68)	403	53	545	42	UST
Yang et al. (81)	150	3	386	6	UST

#### 3.5.2 VDZ vs. ustekinumab

Twelve studies were included in this comparison. Meta-analysis showed that VDZ was associated with a significantly higher overall infection risk compared with ustekinumab (RR = 1.625, 95% CI: 1.324–1.994, *P* < 0.001). Heterogeneity was negligible and not statistically significant (*Q* = 12.20, df = 11, *P* = 0.349; *I*^2^ = 0.26%, tau^2^ = 0.0003) (Table 2 and Supplementary Figure 3).

#### 3.5.3 VDZ vs. placebo

Four studies were included in this comparison. Meta-analysis showed no statistically significant difference in overall infection risk between VDZ and placebo (RR = 1.019, 95% CI: 0.768–1.353, *P* = 0.844). No significant heterogeneity was detected (*Q* = 2.82, df = 3, *P* = 0.420; *I*^2^ = 0.00%, tau^2^ < 0.001) (Table 2 and Supplementary Figure 4).

### 3.6 Subgroup analyses and meta-regression

To explore potential sources of heterogeneity, subgroup analyses were performed according to publication year, study design, study scope/organization type, predominant patient age group, geographic region, and follow-up duration. All analyses employed mixed-effects models with Knapp-Hartung adjustment.

#### 3.6.1 Subgroup by study design

Studies were categorized as randomized controlled trials, prospective cohort studies, retrospective cohort studies, and other designs. The pooled infection incidences were 22.46% (95% CI: 10.77–41.18%) for RCTs, 20.21% (95% CI: 11.47–33.15%) for prospective cohort studies, 9.57% (95% CI: 6.38–14.18%) for retrospective cohort studies, and 24.47% (95% CI: 5.96–63.80%) for other designs. The between-group difference was statistically significant (*P* < 0.001), confirming that study design was a major source of heterogeneity.

#### 3.6.2 Subgroup by geographic region

Studies were categorized by geographic region: Asia, Europe, North America, and Global/multinational. The pooled infection incidences were 7.28% (95% CI: 3.35–15.08%) for Asian studies, 16.39% (95% CI: 10.28–25.14%) for European studies, 11.50% (95% CI: 6.48–19.59%) for North American studies, and 15.26% (95% CI: 7.75–27.85%) for Global studies. The between-group difference was statistically significant (*P* < 0.001), indicating important geographic variation in infection risk.

#### 3.6.3 Subgroup by follow-up duration

Studies were grouped into three predefined categories based on mean or median follow-up duration: short-term ( ≤ 6 months), medium-term (7–12 months), and long-term ( > 12 months). The pooled infection incidences were 13.30% (95% CI: 5.29–29.66%) for short-term studies, 10.54% (95% CI: 6.94–15.70%) for medium-term studies, and 16.97% (95% CI: 10.63–26.01%) for long-term studies. The between-group difference was statistically significant (*P* < 0.001).

Additional subgroup analyses yielded the following results. According to publication year, studies were dichotomized into those published ≤ 2020 and those published > 2020; the two categories showed pooled incidences of 14.03% (95% CI: 9.28–20.67%) and 11.84% (95% CI: 7.70–17.77%), with a significant difference (*P* < 0.001). Subgroup analysis by study scope/organization type included five categories: single-center studies, national multicenter studies, multinational multicenter studies, global or large-scale multicenter studies/databases, and nationwide studies based on administrative or health registry databases; the pooled incidences ranged from 6.70 to 30.22% across these groups (all *P* < 0.001). When stratified by predominant patient age group, studies were classified as adult, pediatric, and elderly; the pooled incidences were 12.19% (95% CI: 8.65%–16.97%), 21.19% (95% CI: 6.78–49.88%), and 13.66% (95% CI: 6.71–25.81%), again with significant between-group heterogeneity (*P* < 0.001). Notably, despite these significant subgroup differences, residual heterogeneity remained substantial across all analyses (residual I^2^ ranging from 95.73 to 97.93%).

#### 3.6.4 Meta-regression analysis

Meta-regression was performed to quantify the contribution of multiple covariates to the observed heterogeneity. The model explained 15.64% of the total between-study variance, although the overall omnibus test did not reach statistical significance [*F*(14, 48) = 1.75, *P* = 0.076]. Among the individual moderators, retrospective cohort study design was significantly associated with lower infection risk compared with RCTs (coefficient = −1.6688, *P* = 0.0133), and European geographic region was associated with significantly higher infection risk relative to Asia (coefficient = 1.2987, *P* = 0.0266). North American region showed a trend toward higher risk, though this did not reach statistical significance (coefficient = 1.1201, *P* = 0.0586). Follow-up duration was positively associated with infection risk, but the effect was not statistically significant (coefficient = 0.3778, *P* = 0.1743). Other factors, including publication year, study scope, and predominant patient age, were not significant moderators in the multivariate model. Given that the overall omnibus test was not statistically significant, the significant individual moderators should be interpreted with caution and regarded as hypothesis-generating rather than confirmatory (Supplementary Figure 5).

## 4 Discussion

This systematic review and meta-analysis, synthesizing data from 66 studies and 24,000 IBD patients, provides a comprehensive and methodologically updated assessment of infection risk associated with vedolizumab. The overall pooled infection incidence was 13.08%, with respiratory tract infections identified as the most common type. Crucially, the inclusion of head-to-head comparative analyses revealed that VDZ has a similar infection risk to TNF inhibitors and placebo, but a significantly higher risk compared to ustekinumab. These findings refine the safety profile of VDZ by moving beyond a single-arm incidence estimate to a comparative framework, which is essential for clinical decision-making.

### 4.1 Interpretation of overall and site-specific infection risk

The overall infection incidence of 13.08% (95% CI: 9.85–17.18%) aligns with the range reported in previous pooled analyses (4, 5), but its interpretation as a standalone clinical benchmark is highly limited. The extremely wide 95% prediction interval (1.30–63.29%) quantitatively demonstrates that the true infection risk in a future, real-world IBD population treated with VDZ could be anywhere from negligible to substantial (15). This extreme between-study variability, rather than the point estimate itself, is the most critical finding from the single-arm analysis. It confirms that the observed “incidence” is not a stable drug-specific property but a composite measure heavily confounded by study design, baseline population risk, and concomitant therapies. Clinically, an unstratified pooled proportion does not inform whether an infection in a VDZ-treated patient is attributable to the drug, to active IBD, to prior immunosuppression, or to their combination.

The site-specific analysis identified respiratory tract infections as the most common event. However, the pooled incidence of 4.25% must be contextualized by its significant heterogeneity (*I*^2^ = 98.24%) and the fact that most events were mild to moderate upper respiratory infections. The identification of digestive system infections (2.18%) as the second most common type is clinically congruent with an IBD population, where distinguishing a disease flare from an enteric infection can be challenging. Critically, systemic invasive, nervous system, and other rare infections showed very low incidences with negligible heterogeneity (*I*^2^ = 0%), representing the most statistically stable and clinically reassuring finding of this analysis. These data support the gut-selective mechanism of VDZ, which is not associated with a systemic pan-immunosuppression signal.

### 4.2 Comparative safety: the clinical value of relative risk

The core clinical question is not the absolute infection rate on VDZ, but how its safety compares to therapeutic alternatives. This analysis directly addresses this by pooling comparative risk ratios. We found no significant difference in overall infection risk between VDZ and TNF inhibitors (RR = 0.908, 95% CI: 0.668–1.234, *P* = 0.513). This reframes the narrative: while TNF inhibitors are associated with a well-known risk of systemic and opportunistic infections, VDZ demonstrates a similar overall infection rate in these studies (82, 83). This result does not mean the two drug classes have identical safety profiles; rather, the non-significant difference may reflect that the dominant driver of common, non-opportunistic infections in both groups is the underlying IBD severity and concomitant steroid use rather than a specific immunosuppressive mechanism. However, no opportunistic infections were reported in the VDZ-exclusive cohorts, which remains a key differentiator from TNF inhibitors, where such events are well-documented.

The most clinically actionable finding is the significantly higher overall infection risk with VDZ compared to ustekinumab (RR = 1.625, 95% CI: 1.324–1.994, *P* < 0.001). This result was highly consistent across studies (*I*^2^ = 0.26%), lending strong credibility to the estimate. This difference may stem from the divergent therapeutic targets: VDZ blocks lymphocyte trafficking, potentially leaving mucosal immune surveillance in the gut partially compromised, whereas UST targets the IL-12/23 pathway with a broader but distinct immunomodulatory profile. This finding provides a concrete, evidence-based data point for clinicians choosing between these two commonly sequenced second-line biologics, especially in patients with a history of recurrent infections.

### 4.3 Exploration of heterogeneity and methodological insights

The extreme heterogeneity (*I*^2^> 95%) across most single-arm analyses is not a limitation to be dismissed but a principal result. Meta-regression and subgroup analyses systematically explored its sources, revealing that study design and geographic region were significant moderators. RCTs reported a pooled infection incidence (22.46%) more than double that of retrospective cohort studies (9.57%). This finding is critical: it strongly suggests that the method of event ascertainment, with active surveillance in RCTs capturing a high frequency of mild, transient events versus passive recording from medical charts in retrospective studies, fundamentally drives the “infection risk” signal. This differential ascertainment makes the pooling of these disparate designs into a single estimate statistically unstable and clinically misleading. Geographic differences, such as lower reported rates in Asian studies (7.28%), likely reflect not only true epidemiologic variation in infectious disease burden but also differences in healthcare monitoring practices and cultural thresholds for diagnosing and reporting mild adverse events.

### 4.4 Vaccination, pre-treatment screening, and regional considerations

The geographic heterogeneity identified has direct clinical implications for infection prevention strategies. The significant variation in background infection epidemiology, particularly for respiratory and latent infections like tuberculosis, means that pre-treatment screening and vaccination protocols must be adapted to regional guidelines, not a single global standard (84). While this review confirms that VDZ itself is not associated with an elevated risk of opportunistic infections like tuberculosis, a patient’s underlying IBD, nutritional status, and potential for future steroid use necessitate that standard vaccination schedules (e.g., influenza, pneumococcal, recombinant zoster vaccine) be optimized before initiating VDZ therapy. The low signal for systemic infections suggests that VDZ may be a suitable choice in a patient who is a candidate for biologic therapy but has a high baseline risk for tuberculosis reactivation, provided their vaccination and screening are up-to-date. These real-world considerations are integral to translating the pooled data into individualized care.

### 4.5 Strengths, limitations, and clinical interpretation

This study’s strengths include a rigorous methodology employing the Hartung-Knapp adjustment and prediction intervals, a large sample size, and a clinically informative comparative framework. However, the single-arm incidence estimates carry a high risk of being misinterpreted as precise clinical benchmarks. Furthermore, the restriction to English-language publications may introduce language bias, potentially omitting relevant studies published in other languages and limiting the global representativeness of the aggregated results. The substantial heterogeneity, coupled with the methodological observation that study design accounts for a large proportion of the observed variance, precludes the uncritical adoption of a single aggregate point estimate (such as 13.08%) as a guideline-level benchmark for vedolizumab-associated infection risk. A more clinically rigorous and scientifically defensible interpretation is that mild, self-limited infections, particularly of the respiratory tract, are common and occur at a frequency broadly comparable to that observed with other first- and second-line biologic agents. By contrast, the incidence of serious, systemic, and opportunistic infections remains low and is not readily distinguishable from the background risk attributable to active inflammatory bowel disease itself. The finding of a higher infection risk versus UST should be presented as preliminary but hypothesis-generating data, rather than definitive evidence, until validated by large, dedicated, real-world comparative studies or RCTs that control for steroid exposure and disease severity.

### 4.6 Clinical recommendations

Based on these findings, clinical recommendations can be refined but should not be overstated. Before initiating VDZ, a standard infection risk assessment should be conducted, including screening for tuberculosis and viral hepatitis per local guidelines, and an evaluation and optimization of vaccination status. During treatment, for high-risk patients, periodic monitoring for respiratory and other infectious symptoms is appropriate, with routine blood and inflammatory markers checked as clinically indicated. In the event of a severe infection, temporary suspension of VDZ is a reasonable clinical strategy. The significantly higher infection signal compared to UST, while not definitive, suggests that clinicians should carefully weigh the therapeutic benefits and safety profiles of all available agents, especially in patients with a history of recurrent or complicated infections. The decision should always be individualized, avoiding unnecessary combination with systemic corticosteroids or immunosuppressants whenever possible.

### 4.7 Future research directions

Future research must abandon the pooling of single-arm infection rates as a primary aim. The priority should be large-scale, propensity-score-matched or prospective comparative studies that adjust for time-varying confounders like disease activity and steroid use to isolate the drug-specific infection signal. Head-to-head safety comparisons with newer agents like JAK inhibitors and IL-23p19 inhibitors are critical to define the modern therapeutic sequence. Longer-term surveillance studies are needed to confirm the low risk of rare and opportunistic infections. Finally, a shift from reporting crude incidence proportions to person-year adjusted incidence rates with standardized outcome definitions (e.g., CTCAE) would substantially reduce methodological noise and produce clinically digestible risk estimates (9).

## 5 Conclusion

In this meta-analysis of 66 studies and 24,000 patients, vedolizumab-treated IBD patients had a 13.08% pooled overall infection incidence, dominated by mild respiratory tract infections, while serious systemic and opportunistic infections were rare. Comparative analyses showed that VDZ carries a similar infection risk to TNF inhibitors and placebo, but a significantly higher risk compared to ustekinumab. The extreme heterogeneity and wide prediction intervals indicate that the single-arm pooled incidence should not be interpreted as a definitive drug-specific risk estimate, and that infection risk assessment in VDZ-treated patients must be individualized within a comparative framework.

## Data Availability

The original contributions presented in the study are included in the article/Supplementary material, further inquiries can be directed to the corresponding authors.

## Supplementary material

Supplementary Figure 1 Funnel plot of overall infection risk in VDZ-Treated IBD patients.

Supplementary Figure 2 Forest plot of risk ratios for overall infection comparing VDZ vs. TNFi.

Supplementary Figure 3 Forest plot of risk ratios for overall infection comparing VDZ vs. UST.

Supplementary Figure 4 Forest plot of risk ratios for overall infection comparing VDZ vs. PBO.

Supplementary Figure 5 Forest plot of meta-regression analysis for sources of heterogeneity in overall infection risk comparison.
