# Supplementary material for: Infection risk in inflammatory bowel disease patients treated with vedolizumab: a systematic review and meta-analysis
Source: Front Med (Lausanne). 2026 Jun 11;13:1806488. doi: 10.3389/fmed.2026.1806488 (PMC13293795; doi:10.3389/fmed.2026.1806488)
Supplement: Supplementary file 1 [file Table_1.docx]

| **Supplementary Table 1. Classification of Infection Types Included in the Study** | |
| --- | --- |
| Infection Term as Reported in Included Studies | Infection Category |
| Pneumonia | Respiratory tract infection |
| Lung infection | Respiratory tract infection |
| Upper respiratory tract infection | Respiratory tract infection |
| Nasopharyngitis | Respiratory tract infection |
| Pharyngitis | Respiratory tract infection |
| Rhinitis | Respiratory tract infection |
| Sinusitis | Respiratory tract infection |
| Sinopulmonary infection | Respiratory tract infection |
| Nasosinopulmonary infection | Respiratory tract infection |
| Bronchitis | Respiratory tract infection |
| Lower respiratory tract infection | Respiratory tract infection |
| Influenza or influenza-like illness | Respiratory tract infection |
| Influenza-like symptoms | Respiratory tract infection |
| Common cold | Respiratory tract infection |
| Ear, nose, and throat infection | Respiratory tract infection |
| Otitis media | Respiratory tract infection |
| Ear infection | Respiratory tract infection |
| Peritonsillar abscess | Respiratory tract infection |
| Pneumonia/pneumothorax (infection-related) | Respiratory tract infection |
| Bronchitis/pneumonia (combined) | Respiratory tract infection |
| Nasopharyngitis/upper respiratory tract infection (combined) | Respiratory tract infection |
| Upper and lower respiratory tract infection (combined) | Respiratory tract infection |
| Gastrointestinal infection | Gastrointestinal infection |
| Gastroenteritis | Gastrointestinal infection |
| Infectious gastroenteritis | Gastrointestinal infection |
| Infectious diarrhea | Gastrointestinal infection |
| Diarrhea (infectious) | Gastrointestinal infection |
| Enteric infection | Gastrointestinal infection |
| Infectious enteritis | Gastrointestinal infection |
| Clostridioides difficile infection | Gastrointestinal infection |
| Clostridioides difficile colitis | Gastrointestinal infection |
| Cytomegalovirus colitis | Gastrointestinal infection |
| Helicobacter pylori gastritis | Gastrointestinal infection |
| Colitis | Gastrointestinal infection |
| Campylobacter jejuni infection | Gastrointestinal infection |
| Salmonella gastroenteritis | Gastrointestinal infection |
| Norovirus infection | Gastrointestinal infection |
| Strongyloides infection | Gastrointestinal infection |
| Campylobacter infection | Gastrointestinal infection |
| Salmonella infection | Gastrointestinal infection |
| Giardiasis | Gastrointestinal infection |
| Cholangitis | Gastrointestinal infection |
| Abdominal abscess | Gastrointestinal infection |
| Mesenteric abscess | Gastrointestinal infection |
| Intra-abdominal abscess | Gastrointestinal infection |
| Abdominal and gastrointestinal infection (combined) | Gastrointestinal infection |
| Urinary tract infection | Genitourinary system infection |
| Pyelonephritis | Genitourinary system infection |
| Cystitis | Genitourinary system infection |
| Urinary tract infection | Genitourinary system infection |
| Skin infection | Skin and soft tissue infection |
| Soft tissue infection | Skin and soft tissue infection |
| Cellulitis | Skin and soft tissue infection |
| Abscess (skin) | Skin and soft tissue infection |
| Postoperative wound infection | Skin and soft tissue infection |
| Surgical site infection | Skin and soft tissue infection |
| Injection site infection | Skin and soft tissue infection |
| Vulvar abscess | Skin and soft tissue infection |
| Buttock abscess | Skin and soft tissue infection |
| Skin rash (infectious) | Skin and soft tissue infection |
| Butterfly rash (infectious) | Skin and soft tissue infection |
| Herpes zoster | Skin and soft tissue infection |
| Herpes zoster ophthalmicus | Skin and soft tissue infection |
| Herpes simplex virus reactivation | Skin and soft tissue infection |
| Oral herpes | Skin and soft tissue infection |
| Cold sore / Herpes labialis | Skin and soft tissue infection |
| Varicella | Skin and soft tissue infection |
| Pityriasis versicolor | Skin and soft tissue infection |
| Hand, foot and mouth disease | Skin and soft tissue infection |
| Skin and soft tissue infection (combined) | Skin and soft tissue infection |
| Septicemia | Systemic/invasive infection |
| Bacteremia | Systemic/invasive infection |
| Sepsis | Systemic/invasive infection |
| Catheter-related bloodstream infection | Systemic/invasive infection |
| Gram-negative bacillus bacteremia | Systemic/invasive infection |
| Pelvic sepsis | Systemic/invasive infection |
| Bloodstream infection | Systemic/invasive infection |
| Osteomyelitis | Systemic/invasive infection |
| Meningitis | Nervous system infection |
| Encephalitis | Nervous system infection |
| Meningoencephalitis | Nervous system infection |
| Transverse myelitis | Nervous system infection |
| Musculoskeletal infection | Musculoskeletal system infection |
| Conjunctivitis | Ocular infection |
| Tuberculosis | Other specific pathogen infection |
| Pulmonary tuberculosis | Other specific pathogen infection |
| Tuberculosis reactivation | Other specific pathogen infection |
| Cytomegalovirus infection (non-colonic) | Other specific pathogen infection |
| Cytomegalovirus reactivation | Other specific pathogen infection |
| Epstein-Barr virus infection | Other specific pathogen infection |
| COVID-19 infection | Other specific pathogen infection |
| SARS-CoV-2 infection | Other specific pathogen infection |
| Histoplasmosis | Other specific pathogen infection |
| Aspergillosis | Other specific pathogen infection |
| Bronchopulmonary aspergillosis | Other specific pathogen infection |
| Disseminated histoplasmosis | Other specific pathogen infection |
| Pneumocystis jirovecii pneumonia | Other specific pathogen infection |
| Listeriosis | Other specific pathogen infection |
| Listeria meningitis | Other specific pathogen infection |
| Atypical mycobacterial infection | Other specific pathogen infection |
| Esophageal candidiasis | Other specific pathogen infection |
| Oral candidiasis | Other specific pathogen infection |
| Viral infection (unspecified) | Other specific pathogen infection |
| Fungal infection (unspecified) | Other specific pathogen infection |
| Bacterial infection (unspecified) | Other specific pathogen infection |
| Fever of unknown origin | Other/unspecified infection |
| Fever | Other/unspecified infection |
| Unspecified infection | Other/unspecified infection |
| Other infection | Other/unspecified infection |
| Infection not otherwise specified | Other/unspecified infection |
| Infection (unclassified) | Other/unspecified infection |
| Infectious complication | Other/unspecified infection |
| Infectious event | Other/unspecified infection |
