# Supplementary material for: Infection risk in inflammatory bowel disease patients treated with vedolizumab: a systematic review and meta-analysis
Source: Front Med (Lausanne). 2026 Jun 11;13:1806488. doi: 10.3389/fmed.2026.1806488 (PMC13293795; doi:10.3389/fmed.2026.1806488)
Supplement: Supplementary file 2 [file Supplementary_file_2.docx]

**Search expression**

**PubMed:**

Search: ((vedolizumab) OR (Anti-alpha 4 beta 7 integrin antibody)) AND ((Inflammatory Bowel Disease) OR (Crohn's Disease) OR (Ulcerative Colitis)) AND ((Infection) OR (Infectious complication)) Sort by: Publication Date

("vedolizumab"[Supplementary Concept] OR "vedolizumab"[All Fields] OR ("Anti-alpha"[All Fields] AND "4"[All Fields] AND ("integrin beta7"[Supplementary Concept] OR "integrin beta7"[All Fields] OR "beta 7 integrin"[All Fields]) AND ("antibodie"[All Fields] OR "antibodies"[Supplementary Concept] OR "antibodies"[All Fields] OR "antibodies"[MeSH Terms] OR "antibody s"[All Fields] OR "antibodys"[All Fields] OR "immunoglobulins"[Supplementary Concept] OR "immunoglobulins"[All Fields] OR "antibody"[All Fields] OR "immunoglobulins"[MeSH Terms]))) AND ("inflammatory bowel diseases"[MeSH Terms] OR ("inflammatory"[All Fields] AND "bowel"[All Fields] AND "diseases"[All Fields]) OR "inflammatory bowel diseases"[All Fields] OR ("inflammatory"[All Fields] AND "bowel"[All Fields] AND "disease"[All Fields]) OR "inflammatory bowel disease"[All Fields] OR ("crohn disease"[MeSH Terms] OR ("crohn"[All Fields] AND "disease"[All Fields]) OR "crohn disease"[All Fields] OR ("crohn s"[All Fields] AND "disease"[All Fields]) OR "crohn s disease"[All Fields]) OR ("colitis, ulcerative"[MeSH Terms] OR ("colitis"[All Fields] AND "ulcerative"[All Fields]) OR "ulcerative colitis"[All Fields] OR ("ulcerative"[All Fields] AND "colitis"[All Fields]))) AND ("infect"[All Fields] OR "infectability"[All Fields] OR "infectable"[All Fields] OR "infectant"[All Fields] OR "infectants"[All Fields] OR "infected"[All Fields] OR "infecteds"[All Fields] OR "infectibility"[All Fields] OR "infectible"[All Fields] OR "infecting"[All Fields] OR "infection s"[All Fields] OR "infections"[MeSH Terms] OR "infections"[All Fields] OR "infection"[All Fields] OR "infective"[All Fields] OR "infectiveness"[All Fields] OR "infectives"[All Fields] OR "infectivities"[All Fields] OR "infects"[All Fields] OR "pathogenicity"[MeSH Subheading] OR "pathogenicity"[All Fields] OR "infectivity"[All Fields] OR (("infectious"[All Fields] OR "infectiousness"[All Fields]) AND ("complicances"[All Fields] OR "complicate"[All Fields] OR "complicated"[All Fields] OR "complicates"[All Fields] OR "complicating"[All Fields] OR "complication"[All Fields] OR "complication s"[All Fields] OR "complications"[MeSH Subheading] OR "complications"[All Fields])))

Translations

vedolizumab: "vedolizumab"[Supplementary Concept] OR "vedolizumab"[All Fields]

beta 7 integrin: "integrin beta7"[Supplementary Concept] OR "integrin beta7"[All Fields] OR "beta 7 integrin"[All Fields]

antibody: "antibodie"[All Fields] OR "antibodies"[Supplementary Concept] OR "antibodies"[All Fields] OR "antibodies"[MeSH Terms] OR "antibody's"[All Fields] OR "antibodys"[All Fields] OR "immunoglobulins"[Supplementary Concept] OR "immunoglobulins"[All Fields] OR "antibody"[All Fields] OR "immunoglobulins"[MeSH Terms]

Inflammatory Bowel Disease: "inflammatory bowel diseases"[MeSH Terms] OR ("inflammatory"[All Fields] AND "bowel"[All Fields] AND "diseases"[All Fields]) OR "inflammatory bowel diseases"[All Fields] OR ("inflammatory"[All Fields] AND "bowel"[All Fields] AND "disease"[All Fields]) OR "inflammatory bowel disease"[All Fields]

Crohn's Disease: "crohn disease"[MeSH Terms] OR ("crohn"[All Fields] AND "disease"[All Fields]) OR "crohn disease"[All Fields] OR ("crohn's"[All Fields] AND "disease"[All Fields]) OR "crohn's disease"[All Fields]

Ulcerative Colitis: "colitis, ulcerative"[MeSH Terms] OR ("colitis"[All Fields] AND "ulcerative"[All Fields]) OR "ulcerative colitis"[All Fields] OR ("ulcerative"[All Fields] AND "colitis"[All Fields])

Infection: "infect"[All Fields] OR "infectability"[All Fields] OR "infectable"[All Fields] OR "infectant"[All Fields] OR "infectants"[All Fields] OR "infected"[All Fields] OR "infecteds"[All Fields] OR "infectibility"[All Fields] OR "infectible"[All Fields] OR "infecting"[All Fields] OR "infection's"[All Fields] OR "infections"[MeSH Terms] OR "infections"[All Fields] OR "infection"[All Fields] OR "infective"[All Fields] OR "infectiveness"[All Fields] OR "infectives"[All Fields] OR "infectivities"[All Fields] OR "infects"[All Fields] OR "pathogenicity"[Subheading] OR "pathogenicity"[All Fields] OR "infectivity"[All Fields]

Infectious: "infectious"[All Fields] OR "infectiousness"[All Fields]

complication: "complicances"[All Fields] OR "complicate"[All Fields] OR "complicated"[All Fields] OR "complicates"[All Fields] OR "complicating"[All Fields] OR "complication"[All Fields] OR "complication's"[All Fields] OR "complications"[Subheading] OR "complications"[All Fields]

**EMBASE:**

#1 (vedolizumab) OR (Anti-alpha 4 beta 7 integrin antibody)

#2 (Inflammatory Bowel Disease) OR (Crohns Disease) OR (Ulcerative Colitis)

#3 (Infection) OR (Infectious complication)

#4 #1 AND #2 AND #3

('vedolizumab'/exp OR vedolizumab OR 'anti-alpha 4 beta 7 integrin antibody' OR ('anti alpha' AND 4 AND beta AND ('7'/exp OR 7) AND ('integrin'/exp OR integrin) AND ('antibody'/exp OR antibody))) AND ('inflammatory bowel disease'/exp OR 'inflammatory bowel disease' OR (inflammatory AND ('bowel'/exp OR bowel) AND ('disease'/exp OR disease)) OR 'crohns disease'/exp OR 'crohns disease' OR (crohns AND ('disease'/exp OR disease)) OR 'ulcerative colitis'/exp OR 'ulcerative colitis' OR (ulcerative AND ('colitis'/exp OR colitis))) AND ('infection'/exp OR infection OR 'infectious complication'/exp OR 'infectious complication' OR (infectious AND ('complication'/exp OR complication)))

**WOS：**

"inflammatory bowel disease" OR "inflammatory bowel diseases" OR IBD OR "Crohn disease" OR "Crohn's disease" OR Crohn OR "ulcerative colitis" OR "UC" (All Fields) and vedolizumab OR entylia OR mln0002 OR "anti-α4β7" OR "anti α4β7" OR "anti-a4b7" OR "anti a4b7" OR "α4β7 integrin" OR "integrin antagonist" (All Fields) and infections OR "infectious complication" OR "serious infection" OR "severe infection" OR "opportunistic infection" OR sepsis OR septicemia OR pneumonia OR tuberculosis OR "herpes zoster" OR "C. difficile" OR "Clostridioides difficile" OR "Clostridium difficile" (All Fields) | 442 results

**OVID：**

("inflammatory bowel disease" OR "inflammatory bowel diseases" OR IBD OR "Crohn disease" OR "Crohn's disease" OR Crohn OR "ulcerative colitis" OR "UC") AND ((vedolizumab OR Entyvio OR MLN0002 OR "anti-α4β7" OR "anti α4β7" OR "anti-a4b7" OR "anti a4b7" OR "α4β7 integrin" OR "integrin antagonist")) AND (infection OR infections OR "infectious complication" OR "serious infection" OR "severe infection" OR "opportunistic infection" OR sepsis OR septicemia OR pneumonia OR tuberculosis OR "herpes zoster" OR "C. difficile" OR "Clostridioides difficile" OR "Clostridium difficile") {Including Limited Related Terms}

**Cochrane Library:**

"inflammatory bowel disease" OR "inflammatory bowel diseases" OR IBD OR "Crohn disease" OR "Crohn's disease" OR Crohn OR "ulcerative colitis" OR "UC" in Title Abstract Keyword AND vedolizumab OR Entyvio OR MLN0002 OR "anti-α4β7" OR "anti α4β7" OR "anti-a4b7" OR "anti a4b7" OR "α4β7 integrin" OR "integrin antagonist" in Title Abstract Keyword AND infection OR infections OR "infectious complication" OR "serious infection" OR "severe infection" OR "opportunistic infection" OR sepsis OR septicemia OR pneumonia OR tuberculosis OR "herpes zoster" OR "C. difficile" OR "Clostridioides difficile" OR "Clostridium difficile" in Title Abstract Keyword - (Word variations have been searched)
