# Supplementary material for: Infection risk in inflammatory bowel disease patients treated with vedolizumab: a systematic review and meta-analysis
Source: Front Med (Lausanne). 2026 Jun 11;13:1806488. doi: 10.3389/fmed.2026.1806488 (PMC13293795; doi:10.3389/fmed.2026.1806488)
Supplement: Supplementary file 3 [file Table_3.docx]

**Supplementary Table 3.1** The NOS scores of the included studies

| Study ID | Q1 | Q2 | Q3 | Q4 | Q5 | Q6 | Q7 | Q8 | NOS score selection (out of 4) | NOS score comparability (out of 2) | NOS score outcome (out of 3) | NOS total score (out of 9) |
| --- | --- | --- | --- | --- | --- | --- | --- | --- | --- | --- | --- | --- |
| Adar2019 | 1 | 1 | 0 | 1 | 2 | 1 | 0 | 1 | 3 | 2 | 2 | 7 |
| Alric2020 | 1 | 0 | 1 | 1 | 2 | 1 | 0 | 1 | 3 | 2 | 2 | 7 |
| Amiot2019_3y | 1 | 1 | 1 | 1 | 0 | 1 | 0 | 1 | 4 | 0 | 2 | 6 |
| Amiot2026 | 1 | 1 | 1 | 0 | 2 | 1 | 0 | 1 | 3 | 2 | 2 | 7 |
| Asscher2020 | 1 | 1 | 0 | 1 | 2 | 1 | 0 | 1 | 3 | 2 | 2 | 7 |
| Barberio2022 | 1 | 1 | 1 | 1 | 2 | 1 | 0 | 1 | 4 | 2 | 2 | 8 |
| Baumgart2016 | 1 | 1 | 1 | 1 | 0 | 1 | 0 | 1 | 4 | 0 | 2 | 6 |
| Biemans2020 | 1 | 0 | 1 | 1 | 1 | 1 | 0 | 1 | 3 | 1 | 2 | 6 |
| Bohm2020 | 0 | 1 | 1 | 1 | 2 | 1 | 0 | 1 | 3 | 2 | 2 | 7 |
| Bor2020 | 1 | 1 | 1 | 1 | 0 | 1 | 0 | 1 | 4 | 0 | 2 | 6 |
| Bozon2023 | 1 | 1 | 1 | 1 | 2 | 1 | 0 | 1 | 4 | 2 | 2 | 8 |
| Buer2019 | 1 | 1 | 1 | 1 | 0 | 1 | 0 | 1 | 4 | 0 | 2 | 6 |
| Burgevin2022 | 1 | 1 | 0 | 1 | 2 | 1 | 0 | 1 | 3 | 2 | 2 | 7 |
| Chaparro2018 | 1 | 1 | 1 | 1 | 1 | 1 | 0 | 1 | 4 | 1 | 2 | 7 |
| Choi2022 | 0 | 1 | 1 | 0 | 1 | 1 | 0 | 1 | 3 | 1 | 2 | 6 |
| Christensen2018 | 1 | 0 | 1 | 1 | 1 | 1 | 0 | 1 | 3 | 1 | 2 | 6 |
| Cohen2020 | 1 | 1 | 1 | 1 | 2 | 1 | 1 | 1 | 4 | 2 | 3 | 9 |
| Colombel2017 | 1 | 1 | 1 | 1 | 2 | 1 | 1 | 1 | 4 | 2 | 3 | 9 |
| Conrad2016 | 1 | 1 | 1 | 1 | 0 | 1 | 1 | 1 | 4 | 0 | 3 | 7 |
| Dalal2023 | 1 | 1 | 1 | 1 | 2 | 1 | 1 | 1 | 4 | 2 | 3 | 9 |
| Danese2021 | 1 | 1 | 1 | 1 | 0 | 1 | 1 | 1 | 4 | 0 | 3 | 7 |
| Dar2024 | 0 | 1 | 1 | 1 | 1 | 1 | 1 | 0 | 3 | 1 | 2 | 6 |
| DArcangelo2021 | 1 | 1 | 1 | 1 | 0 | 1 | 1 | 1 | 4 | 0 | 3 | 7 |
| Dragoni2019 | 1 | 0 | 1 | 1 | 1 | 1 | 0 | 1 | 3 | 1 | 2 | 6 |
| Fabiszewska2021 | 1 | 1 | 1 | 0 | 1 | 1 | 0 | 1 | 3 | 1 | 2 | 6 |
| Feagan2018 | 0 | 1 | 1 | 1 | 2 | 1 | 1 | 1 | 3 | 2 | 3 | 8 |
| Gebeyehu2023 | 1 | 0 | 1 | 1 | 2 | 1 | 0 | 1 | 3 | 2 | 2 | 7 |
| Holvoet2024 | 1 | 1 | 0 | 1 | 2 | 1 | 0 | 1 | 3 | 2 | 2 | 7 |
| Huang2025 | 1 | 0 | 1 | 1 | 1 | 1 | 0 | 1 | 3 | 1 | 2 | 6 |
| Hupé2020 | 0 | 1 | 1 | 1 | 1 | 1 | 0 | 1 | 3 | 1 | 2 | 6 |
| Kaku2025 | 1 | 1 | 1 | 0 | 2 | 1 | 1 | 0 | 3 | 2 | 2 | 7 |
| Khan2021 | 1 | 1 | 0 | 1 | 1 | 1 | 0 | 1 | 3 | 1 | 2 | 6 |
| Khan2025 | 1 | 1 | 1 | 1 | 2 | 0 | 1 | 1 | 4 | 2 | 2 | 8 |
| Kochar2022 | 1 | 1 | 1 | 1 | 2 | 1 | 1 | 1 | 4 | 2 | 3 | 9 |
| Kopylov2019 | 0 | 1 | 0 | 1 | 1 | 1 | 1 | 0 | 3 | 1 | 2 | 6 |
| Lee2021 | 0 | 1 | 1 | 1 | 1 | 1 | 0 | 1 | 3 | 1 | 2 | 6 |
| Loftus2020 | 1 | 1 | 1 | 1 | 1 | 0 | 1 | 1 | 4 | 1 | 2 | 7 |
| Louis2025 | 1 | 0 | 1 | 1 | 2 | 1 | 0 | 1 | 3 | 2 | 2 | 7 |
| Lukin2022 | 1 | 1 | 1 | 1 | 2 | 1 | 0 | 1 | 4 | 2 | 2 | 8 |
| Mantzaris2024 | 1 | 1 | 1 | 0 | 2 | 1 | 0 | 1 | 3 | 2 | 2 | 7 |
| Meng2024 | 1 | 0 | 1 | 1 | 2 | 1 | 0 | 1 | 3 | 2 | 2 | 7 |
| Meserve2019 | 1 | 0 | 1 | 1 | 1 | 1 | 0 | 1 | 3 | 1 | 2 | 6 |
| Na2024 | 0 | 1 | 1 | 0 | 1 | 1 | 0 | 1 | 3 | 1 | 2 | 6 |
| Novello2020 | 1 | 1 | 1 | 1 | 2 | 0 | 1 | 1 | 4 | 2 | 2 | 8 |
| Pabla2022 | 1 | 1 | 1 | 0 | 1 | 0 | 1 | 1 | 3 | 1 | 2 | 6 |
| Pulusu2020 | 1 | 1 | 1 | 0 | 1 | 1 | 0 | 1 | 3 | 1 | 2 | 6 |
| Roberti2020 | 0 | 1 | 1 | 1 | 1 | 1 | 0 | 1 | 3 | 1 | 2 | 6 |
| Sachar2023 | 0 | 1 | 1 | 1 | 2 | 1 | 0 | 1 | 3 | 2 | 2 | 7 |
| Shashi2020 | 1 | 0 | 1 | 1 | 2 | 1 | 1 | 0 | 3 | 2 | 2 | 7 |
| Shelton2015 | 1 | 1 | 0 | 1 | 1 | 1 | 0 | 1 | 3 | 1 | 2 | 6 |
| Singh2016 | 1 | 0 | 1 | 1 | 1 | 1 | 0 | 1 | 3 | 1 | 2 | 6 |
| Singh2022-2 | 1 | 1 | 1 | 1 | 2 | 1 | 1 | 1 | 4 | 2 | 3 | 9 |
| Singh2023 | 0 | 1 | 1 | 1 | 2 | 1 | 0 | 1 | 3 | 2 | 2 | 7 |
| Stallmach2016 | 1 | 0 | 1 | 1 | 1 | 1 | 1 | 0 | 3 | 1 | 2 | 6 |
| Watanabe2020-1 | 1 | 1 | 1 | 1 | 2 | 1 | 1 | 1 | 4 | 2 | 3 | 9 |
| White2020 | 1 | 0 | 1 | 1 | 1 | 1 | 0 | 1 | 3 | 1 | 2 | 6 |
| Wiken2023 | 0 | 1 | 1 | 1 | 1 | 1 | 1 | 0 | 3 | 1 | 2 | 6 |
| Wils2025 | 0 | 1 | 1 | 1 | 1 | 1 | 0 | 1 | 3 | 1 | 2 | 6 |
| Yang2023 | 1 | 1 | 0 | 1 | 2 | 1 | 0 | 1 | 3 | 2 | 2 | 7 |

**Supplementary Table 3.2** The ROB 2.0 scores of the included studies

| Study ID | Randomization process | Deviations from intended interventions | Missing outcome data | Measurement of the outcome | Selection of the reported result | Overall risk of bias |
| --- | --- | --- | --- | --- | --- | --- |
| Chen2025 | Low risk | Low risk | Low risk | Low risk | Low risk | Low risk |
| Feagan2013 | Low risk | Low risk | Low risk | Low risk | Low risk | Low risk |
| Hyams2022 | Low risk | Some concerns | Low risk | Low risk | Low risk | Some concerns |
| Kobayashi2019 | Low risk | Low risk | Low risk | Some concerns | Low risk | Some concerns |
| Naganuma2025 | Low risk | Some concerns | Low risk | Some concerns | Low risk | Some concerns |
| Sandborn2020 | Low risk | Low risk | Low risk | Low risk | Low risk | Low risk |
| Vermeire2022 | Low risk | Low risk | Low risk | Low risk | Low risk | Low risk |
| Watanabe2020 | Low risk | Low risk | Some concerns | Low risk | Low risk | Some concerns |
