# Supplementary figures and images for: Infection risk in inflammatory bowel disease patients treated with vedolizumab: a systematic review and meta-analysis
Source: Front Med (Lausanne). 2026 Jun 11;13:1806488. doi: 10.3389/fmed.2026.1806488 (PMC13293795; doi:10.3389/fmed.2026.1806488)

## Meta-regression coefficients (VDZ infection)

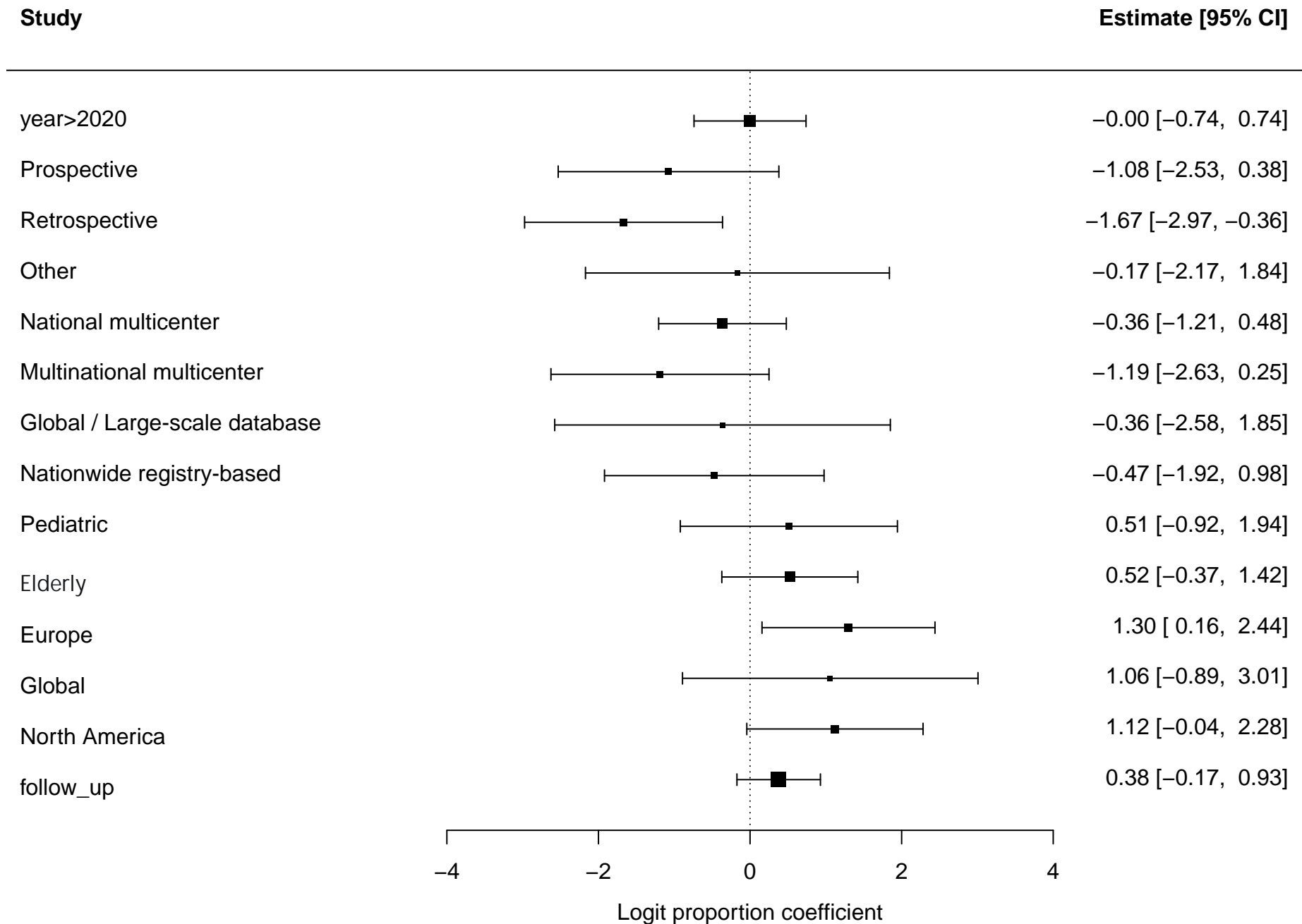

Supplement: Supplementary Figure 1 — Funnel plot of overall infection risk in VDZ-Treated IBD patients. [file Data_Sheet_1.pdf]

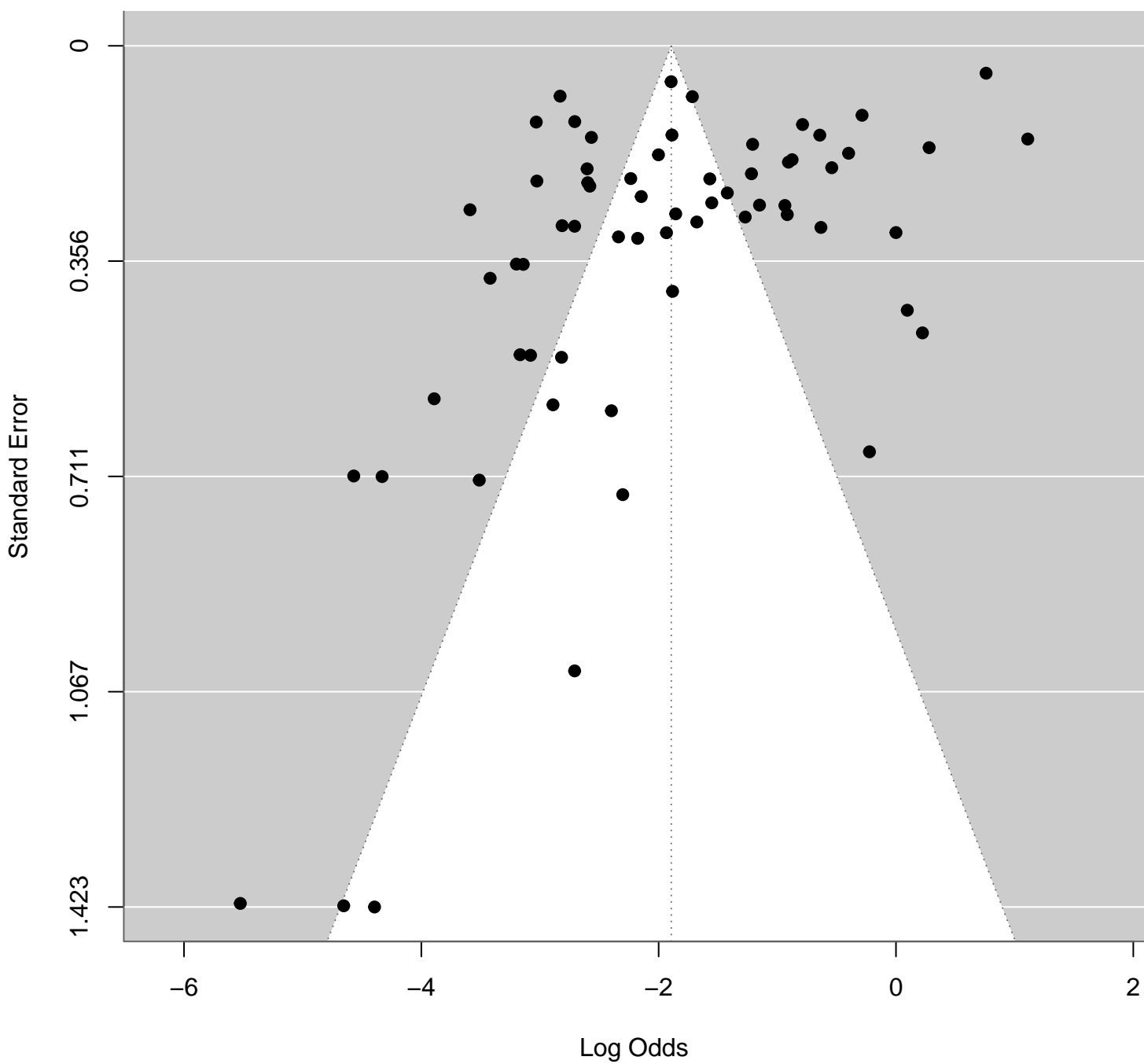

Supplement: Supplementary Figure 2 — Forest plot of risk ratios for overall infection comparing VDZ vs. TNFi. [file Data_Sheet_2.pdf]

VDZ vs UST

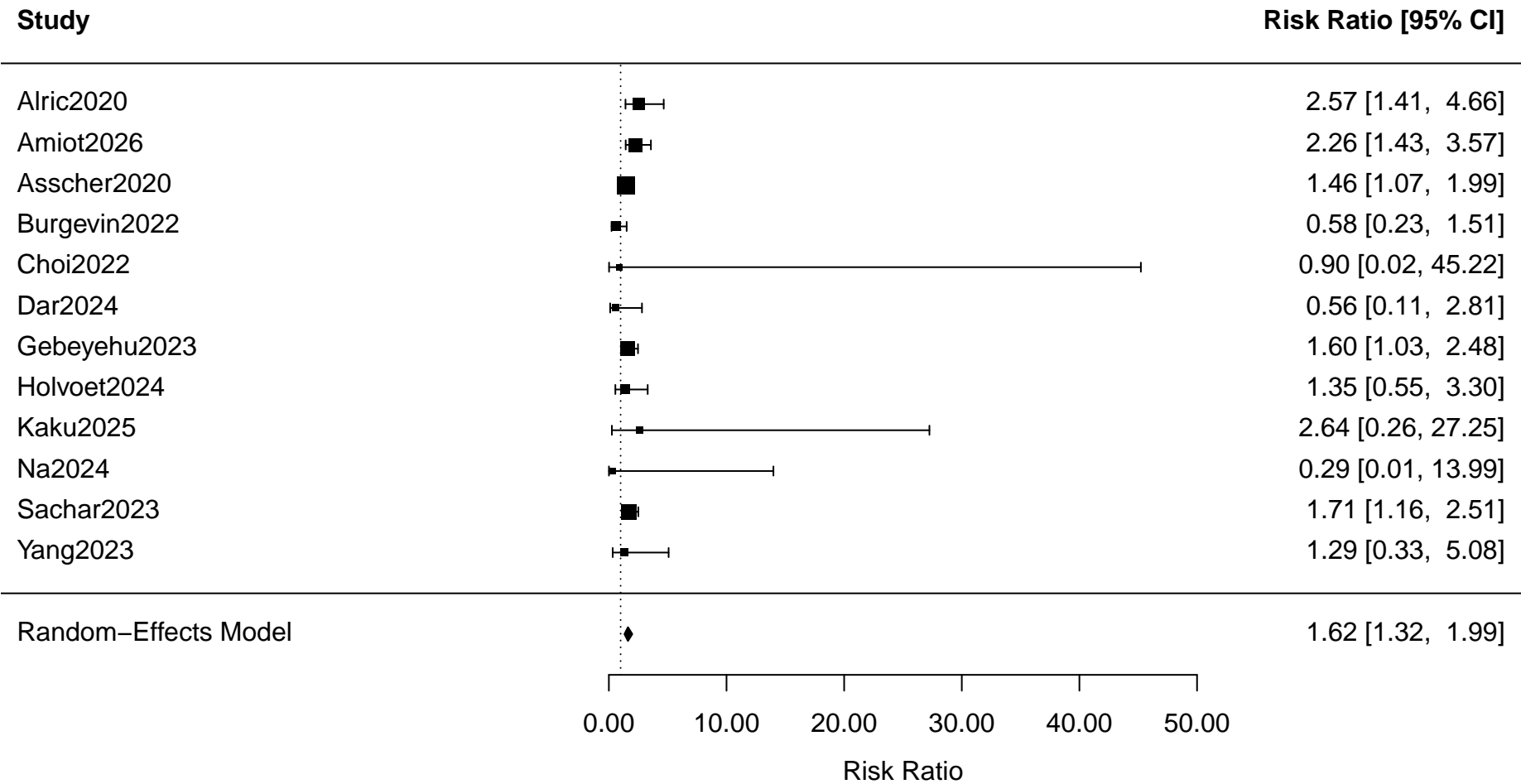

Supplement: Supplementary Figure 3 — Forest plot of risk ratios for overall infection comparing VDZ vs. UST. [file Data_Sheet_3.pdf]

## VDZ vs PBO

**Study**

**Risk Ratio [95% CI]**

Feagan2013

1.03 [0.79, 1.34]

Feagan2018

1.05 [0.59, 1.88]

Vermeire2022

0.91 [0.68, 1.22]

Watanabe2020-1

1.71 [0.87, 3.34]

Random-Effects Model

1.02 [0.77, 1.35]

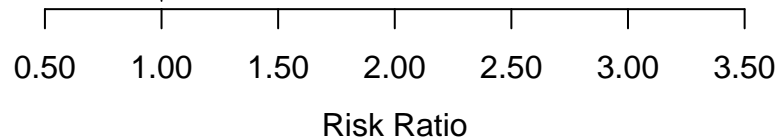

Supplement: Supplementary Figure 4 — Forest plot of risk ratios for overall infection comparing VDZ vs. PBO. [file Data_Sheet_4.pdf]

## VDZ vs TNF

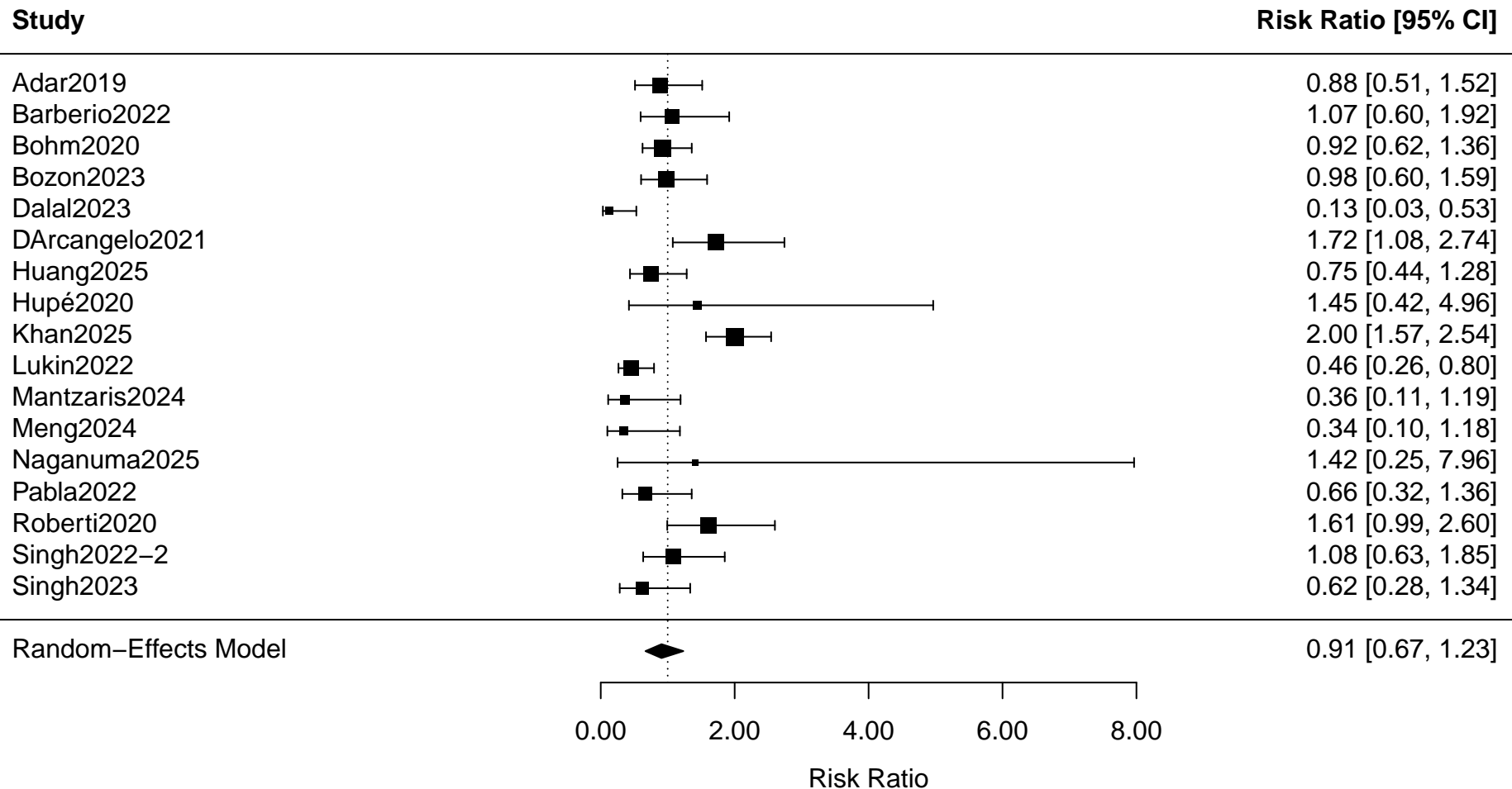

Supplement: Supplementary Figure 5 — Forest plot of meta-regression analysis for sources of heterogeneity in overall infection risk comparison. [file Data_Sheet_5.pdf]
